# Burrow-centered neural model for burrow surveillance in fiddler crabs

**DOI:** 10.1186/1471-2202-12-S1-P168

**Published:** 2011-07-18

**Authors:** Seung-Eun Yu, DaeEun Kim

**Affiliations:** 1 Biological Cybernetics, School of Electrical and Electronic Engineering, Yonsei University, Shinchon, Seoul, 120-749, South Korea

## 

A fiddler crab can estimate how close an intruder is from its burrow [1]. It is believed that crabs use visual information as well as path integration to process that estimation. The fiddler crab *Uca vomeris* defend their burrows against intruders and it seems they use a burrow-centered reference coordinate. The crabs respond when an intruder approaches the burrow within some distance of the burrow. There are several possible cues to estimate the distance between the intruder and the burrow entrance by visual information such as disparity, optical flow, image size and the retinal elevation. However, experiments show only specific visual information is related to the burrow surveillance [1].

The retinal position, azimuth and elevation angle of the intruder in view is related to the distance between the intruder and the observer, following the geometric function. The former parameters can determine the intruder-crab distance depending on the crab-burrow distance. Fiddler crabs on leaving home accumulate the distance and direction of the exploring path can determine the homing distance and direction from the current position to the burrow [2]. To determine the distance between an intruder crab and the burrow entrance, we need to use the crab-burrow distance by the path integration as well as the azimuth and elevation angle of an intruder.

There has been a suggestion that the burrow surveillance in fiddler crabs may be connected with directionally sensitive neurons and distance neurons [1]. Our experiments with a neural model for a mapping function to determine the intruder-burrow distance shows large errors are observed when the intruder is at the opposite side of the crab, since the far vicinity has a low resolution. Thus, we suggest that the fiddler crab might estimate the boundary distance of the response to defend its burrow rather than accurately calculate the distance of the intruder from the burrow. In addition, the crab can determine how directly an intruder is moving toward its burrow. We can thus build a burrow-centered frame of reference with a circular array of directionally sensitive neurons. The firings of the neurons can be mapped from the retinal position of the intruder, that is, the azimuth and elevation angle of the intruder. From that, the fiddler crab can easily determine whether or not the intruder approaches the burrow. As a result, we can build a model of the crab’s response when an intruder approaches the burrow within some distance of the burrow.
